# Effect of Vicarious Fear Learning on Children’s Heart Rate Responses and Attentional Bias for Novel Animals

**DOI:** 10.1037/a0037225

**Published:** 2014-08-25

**Authors:** Gemma Reynolds, Andy P. Field, Chris Askew

**Affiliations:** 1 Department of Psychology, Kingston University; 2 School of Psychology, University of Sussex; 3 Department of Psychology, Kingston University

**Keywords:** childhood anxiety, fear, vicarious learning, modeling

## Abstract

Research with children has shown that vicarious learning can result in changes to 2 of Lang’s (1968) 3 anxiety response systems: subjective report and behavioral avoidance. The current study extended this research by exploring the effect of vicarious learning on physiological responses (Lang’s final response system) and attentional bias. The study used Askew and Field’s (2007) vicarious learning procedure and demonstrated fear-related increases in children’s cognitive, behavioral, and physiological responses. Cognitive and behavioral changes were retested 1 week and 1 month later, and remained elevated. In addition, a visual search task demonstrated that fear-related vicarious learning creates an attentional bias for novel animals, which is moderated by increases in fear beliefs during learning. The findings demonstrate that vicarious learning leads to lasting changes in all 3 of Lang’s anxiety response systems and is sufficient to create attentional bias to threat in children.

Generally, childhood fears are a normal occurrence in childhood and follow a predictive course (Gullone, 2000; Marks, 1987). Despite often being quite intense, fears generally spontaneously recede just as quickly as they appeared; however, for a minority of children, fear persists and is maladaptive to normal functioning (see Muris & Field, 2010, for a review). Fear and anxiety have an adverse impact on children’s social, educational, and emotional functioning (e.g., Pine, 1997; Strauss, Frame, & Forehand, 1987); hence, it is necessary to understand better the acquisition of this psychopathology to inform effective preventative interventions and treatments.

Rachman (1977) suggested three pathways to fear acquisition: (a) direct conditioning during a traumatic event, (b) vicarious learning (learning through observing others), and (c) the transmission of information. All three pathways have been supported empirically (see reviews by Askew & Field, 2008; King, Gullone, & Ollendick, 1998; Merckelbach, de Jong, Muris, & van den Hout, 1996; Muris & Field, 2010). For vicarious learning specifically, research with monkeys (e.g., M. Cook & Mineka, 1987, 1990; Mineka & Cook, 1986, 1988; Mineka, Davidson, Cook, & Keir, 1984; Mineka, Keir, & Price, 1980) and children (e.g., Askew, Dunne, Özdil, Reynolds, & Field, 2013; Askew & Field, 2007; Askew, Kessock-Philip, & Field, 2008; de Rosnay, Cooper, Tsigaras, & Murray, 2006; Dubi, Rapee, Emerton, & Schniering, 2008; Dunne & Askew, 2013; Egliston & Rapee, 2007; Gerull & Rapee, 2002) indicates that it leads to increased fear-related cognitions and avoidance. Evidence from adults also indicates that vicarious learning can lead to changes in skin conductance (Hygge, 1976; Hygge & Öhman, 1978; Olsson & Phelps, 2004). Experimental studies with children have also shown that vicarious learning causes changes in two of Lang’s (1968) fear-related response systems: verbal–cognitive responses (subjective feelings of apprehension) and behavioral responses (avoidance; Askew & Field, 2007). However, it is still unclear whether vicarious learning causes changes in children’s physiological responses, such as heart rate or skin conductance, which may be less vulnerable to demand characteristics than verbal and behavioral responses.

It is well established that children and adults’ self-reported fear or anxiety are commonly associated with physiological changes, such as changes in skin response and blood pressure (Globisch, Hamm, Esteves, & Öhman, 1999) and increased heart rate in feared real or imaginary situations (e.g., E. W. Cook, Melamed, Cuthbert, McNeil, & Lang, 1988; Lang, Levin, Miller, & Kozak, 1983; Lang, Melamed, & Hart, 1970). Heart rate typically increases and decreases in the presence or absence of a feared stimulus (Lang, 1971). For example, Sartory, Rachman, and Grey (1977; see also McNeil, Vrana, Melamed, Cuthbert, & Lang, 1993) explored the relationship between fear and heart rate, and the habituation of heart rate to fear-related stimuli, showing a linear increase in the heart rate of individuals with an animal phobia the closer they were to their feared animal. Weems, Zakem, Costa, Cannon, and Watts (2005) demonstrated that children between the ages of 6 and 17 years showed increased heart rates to a videotape of a large dog (a mildly phobic stimulus) that were consistent with the child’s self-reported anxiety symptoms. They also collected galvanic skin response data, but the association with anxiety was not as strong as between heart rate and anxiety, which they suggested could be because of differences in response latencies to the stimuli. Arguably, skin conductance data are a good measure of aggression, but heart rate is a better measure of fear or anxiety (e.g., Weems et al., 2005).

Given the finding that fear cognitions, avoidance, and physiological changes do not always correlate (Hodgson & Rachman, 1974; Lang et al., 1970; Rachman & Hodgson, 1974; Zinbarg, 1998), it cannot be assumed that because vicarious learning leads to changes in fear beliefs and avoidance behavior, it will also affect physiological responses. Field and Schorah (2007) explored the relationship between heart rate changes and the second of Rachman’s (1977) indirect pathways—verbal information. They found that threat information resulted in not only cognitive and behavioral changes but also physiological changes, compared to a control animal (see also Field & Price-Evans, 2009). If vicarious learning is shown to change children’s heart rates, it would establish that vicarious learning, like the verbal information pathway, causes changes in all three of Lang’s (1968) fear response systems. Moreover, physiological responses are arguably much harder to consciously control than verbal–cognitive and behavioral responses, and may therefore be less prone to demand characteristics. Therefore, this would be particularly convincing evidence that vicarious learning is a viable pathway to fear in children.

Attentional bias refers to the tendency for adults and youths with anxiety to show hyperattention toward threatening or anxiety-related stimuli relative to neutral stimuli (Bar-Haim, Lamy, Pergamin, Bakermans-Kranenburg, & van IJzendoorn, 2007; Cisler & Koster, 2010; Field, Hadwin, & Lester, 2011; Garner, 2010; MacLeod, Mathews, & Tata, 1986; Mathews & MacLeod, 1985; Merckelbach et al., 1996; see Mogg & Bradley, 1998). Information processing biases may play a causal role in anxiety disorders (Mathews & MacLeod, 2002). From a cognitive perspective, high-level information processing biases may also contribute to the maintenance and perpetuation of anxiety by causing stimulus avoidance, and thus preventing the disconfirmation of fear beliefs and the extinction of fear (Field, 2006a; Merckelbach et al., 1996). A wealth of research with clinically anxious children has indicated faster detection of threatening stimuli, and a bias toward processing ambiguous situations or information as threatening (see Field et al., 2011, and Hadwin & Field, 2010, for recent reviews).

One paradigm used frequently to demonstrate attentional biases is the visual search task (e.g., Cisler, Bacon, & Williams, 2009; Flykt, 2005; Hadwin et al., 2003; Lipp, 2006; Lipp, Derakshan, Waters, & Logies, 2004; Miltner, Krieschel, Hecht, Trippe, & Weiss, 2004; Öhman, Flykt, & Esteves, 2001; Rinck, Becker, Kellermann, & Roth, 2003; Rinck, Reinecke, Ellwart, Heuer, & Becker, 2005; Tipples, Young, Quinlan, Broks, & Ellis, 2002; see Donnelly, Hadwin, Manneer, & Richards, 2010, for a review). In this task, participants are asked to locate a target stimulus among a matrix of distracter stimuli. Studies show that participants are faster to locate a threatening stimulus among a matrix of neutral stimuli and slower to locate a neutral stimulus among a matrix of threatening stimuli, as a result of the threatening stimuli capturing attention (Cisler & Koster, 2010). For example, across three experiments using a visual search task displayed on a touch-screen monitor, LoBue and DeLoache (2008) required participants to find a target stimulus (e.g., a snake) among eight distracter stimuli (flowers, frogs, or caterpillars). Both 3-year-olds and adults showed enhanced detection of the threat stimulus.

Field and Purkis’s (2011) conditioning model of fear suggests that associations between a novel conditioned stimulus and a threat outcome (unconditioned stimulus) can be generated directly from vicarious learning and verbal information. Once formed, these associations can drive a variety of different fear-related qualia, such as self-report, physiological responses, attentional processes, and so forth. However, dispositional factors such as trait anxiety will influence the strength of these associations and how quickly they are formed (Field, 2006a, 2006b). With respect to attentional biases, Field and Lester (2010a) have argued that learnt habitual associations are likely to underpin attentional biases, with children gaining more cognitive control over this process as they get older. Field and Lester’s (2010b) review of the literature concludes by suggesting that attentional biases follow a moderation model (see also Kindt & van den Hout, 2001), that is, attentional biases to threat exist in all young children from very early in life and are adaptive. However, their expression is moderated by developmental influences, such as cognitive, social, and emotional development factors. For example, high or low trait anxiety varies across children in different ways, resulting in differences in the maintenance or enhancement of attentional biases. Kindt and van den Hout (2001) suggest that high-anxious children have difficulty inhibiting the innate attentional bias to threat and thus are oversensitive to threat contingencies within their environment.

Several predictions emerge from these models. First, as individual trait anxiety increases, a child should be more susceptible to the learning contingencies related to fear (such as vicarious learning). Second, because high-trait-anxious children have difficulty inhibiting their attentional bias to threat, they should show a greater attentional bias to threat following fear learning (such as vicarious experience). Finally, the extent to which fear-related qualia such as attentional biases are created should be mediated by the effect that learning experiences have (i.e., children who become more fearful after a learning experience should show a greater attentional bias to threat).

There is evidence that these predictions hold up for learning through verbal threat information: Trait anxious children show more avoidance, heart rate change, and attentional bias to a novel animal after threat information (Field, 2006a, 2006b; Field & Price-Evans, 2009), and fear-related responses are mediated by the impact that a learning experience had on subjective ratings for fear (Field & Storkson-Coulson, 2007). However, none of the predictions have been tested for vicarious learning. Thus, the current study aimed to investigate whether fear-related vicarious learning changes children’s heart rate and attentional bias for animals, and to test predictions about the influence of trait anxiety on learning. Further, it is likely that learned responses would need to be fairly robust for actual phobias to develop; therefore, follow-up measures of fear beliefs, avoidance preferences, and attentional bias were taken following vicarious learning, 1 week later and after 1 month.

## Method

### Participants

Forty-four children were recruited from a school in Essex, United Kingdom (21 boys and 23 girls). Typical of longitudinal research, there was some dropout because of absenteeism at follow-ups. All 44 children participated at Time 1, 41 children participated at 1-week follow-up, and 37 children participated at 1-month follow-up. Informed parental consent was obtained 2 weeks prior to the research, and only children with signed consent forms were able to participate. Children also gave verbal assent before taking part. The age range of children was 7 to 9 years, with a mean of 8 years 5.2 months (*SD* = 9.31 months), as normative fears are thought to concentrate on animals around this age (Field & Davey, 2001; Öst, 1987).

### Materials

The experiment was an automated procedure, written in E-Prime 2.0 by the first author, using a Samsung RF511 Laptop and a ProLite T2451MTS 24-in. touch-screen monitor.

#### Questionnaire measures

##### State–Trait Anxiety Inventory for Children (STAIC; Spielberger, Gorsuch, Lushene, Vagg, & Jacobs, 1973)

The STAIC was used to measure children’s state (how the child is feeling “right now”) and trait anxiety (how the child “usually feels”). The scale is comprised of 20 items, and children respond on a 3-point Likert-type scale. Spielberger et al. (1973) report Cronbach’s alphas of .78 for males and .81 for females, and test–retest reliability coefficients of .65 for males and .71 for females.

##### Multidimensional Anxiety Scale for Children – 10 (MASC-10; March, Parker, Sullivan, Stallings, & Conners, 1997)

The MASC-10 was also used to measure children’s anxiety symptoms and trait anxiety (how the child has been “thinking, feeling, or acting recently”). Children respond on a 4-point Likert-type scale (*never true about me*, *rarely true about me*, *sometimes true about me*, and *often true about me*). March et al. (1997) report Cronbach’s alphas of .87 for males and .89 for females.

##### Fear Beliefs Questionnaire (FBQ)

An FBQ (Field & Lawson, 2003) was used to measure children’s fear-related beliefs for the animals. The questionnaire was computer-based and contained seven questions (four that were reverse scored) for each animal (21 questions in total), for example, “Would you go up to a quokka/cuscus/quoll if you saw one?” and “Would you keep your distance from a quokka/cuscus/quoll if you saw one?” Children responded on a 5-point Likert response scale (0 = *no, not at all*; 1 = *no, not really*; 2 = *don’t know/neither*; 3 = *yes, probably*; 4 = *yes, definitely*). The question was displayed at the top of the screen in bold lettering, with a picture of the animal in question in the center of the screen, and the Likert response scale at the bottom of the screen. Children touched the screen to indicate their response. The FBQ was implemented before and after the vicarious learning procedure at Time 1, to explore whether fear-related beliefs changed as a result of vicarious learning, and again at 1-week and 1-month follow-up. Internal consistency was high; before learning, Cronbach’s alpha = .73 (Quokka subscale), .74 (Cuscus subscale), and .83 (Quoll subscale); after learning, alpha = .81, .83, and .88, respectively; at 1-week follow-up, alpha = .78, .86, and .87, respectively; and at 1-month follow-up, alpha = .91, .90, and .88, respectively.

#### Experimental paradigms

##### Vicarious learning

As in previous research (e.g., Askew & Field, 2007), 10 color photographs (approximately 346 × 444 pixels) of three Australian marsupials—a quokka, quoll, and cuscus—were used during vicarious learning as U.K. children were unlikely to have prior experience or fear expectations for the animals. The procedure displayed 30 trials in which a random picture of one animal (e.g., a quokka) was presented alongside a happy face (“happy-paired”), a random picture of a second animal (e.g., a cuscus) was presented alongside a fearful face (“scared-paired”), and a random picture of the third animal (e.g., a quoll) was presented alone (“unpaired”). Unthreatening images of animals in natural positions were selected. Faces used were 20 portrait photographs (also 346 × 444 pixels) of smiling and fearful faces (10 of each: five females and five males) taken from the NimStim Face Stimulus Set (Tottenham, Borscheid, Ellertsen, Marcus, & Nelson, 2002).

The procedure was counterbalanced so that the quokka, quoll, and cuscus were each paired with happy, scared, or no faces in every possible combination. Animals always appeared on the left side of the screen and faces on the right. Based on the parameters used in Askew and Field (2007) and Askew et al. (2008), the picture of the animal appeared on the screen first for 1 s, followed by the picture of the face, and both remained on the screen for another 1 s. Thus, the total length of time for a single trial was 2 s. Each pairing appeared in a random order for each child. The interval between each pairing varied randomly between 2 s and 4 s.

##### Visual search task

A visual search task, created in E-Prime 2.0 by the first author, was used to measure attentional bias toward the animals. Children were asked to indicate as quickly as possible whether a target stimulus was present within a 3 × 3 grid of distracters. That is, they were asked to find one target animal (either a quokka, quoll, or cuscus) among eight leaves distracters, or one leaves target among eight animal distracters. Thus, nine photographs were displayed in each trial. Twenty-four color photographs were selected for each stimulus category. Leaves were selected as distracters to ensure ecological validity. Six stimulus categories were used: the three types of marsupial and three piles of leaves. The three leaf-pile categories contained the same pictures for each animal, but the brightness and hue of the pictures were adjusted in each case so that the saliency of a particular set of distracter leaves matched that of the animal they were presented together with. Three adults and three children, blind to the purpose of the research, were asked to rate the brightness of the pictures. All pictures were rated on a scale from 1 (*very bright*) to 5 (*very dull*). Average ratings were fairly similar across stimuli: 2.79 (*SD* = 0.62) for the cuscus and 2.63 (*SD* = 0.81) for the associated distracter leaves, *t*(5) = 1.25, *p* = .27, *r* = .49; 1.01 (*SD* = 0.57) for the quokka and 1.12 (*SD* = 0.72) for the distracter leaves, *t*(5) = 1.75, *p* = .14, *r* = .62; and 2.48 (*SD* = 1.10) for the quoll and 2.63 (*SD* = 0.76) for the quoll distracter leaves, *t*(5) = 0.44, *p* = .68, *r* = .19. Although differences in ratings were nonsignificant, this may have been because of lack of power given effect sizes were relatively large.

The overall matrix size was 48 × 27 cm, with 1.5 cm between rows and 2.5 cm between columns. The pictures each measured 14 × 8 cm. Directly in front of the monitor was an outline of two child’s handprints on the table and children were asked to place their hands on the prints before each trial to ensure accurate reaction time (RT) data. The task was modified in the same way as LoBue and DeLoache’s (2008) task to ensure the procedure was appropriate for young children. A touch-screen monitor was used, and children were asked to touch the screen as quickly as possible to indicate the presence of a target. Also, in line with LoBue and DeLoache’s (2008) work, a target was always present because the use of a touch-screen monitor prevented the use of no-target trials. To make the task more age appropriate and enjoyable for the children, the task was designed in the form of a game in which a fictional cartoon character, “Safari Sam,” required the children’s help to find his missing items.

The experimenter sat next to the child throughout this task. Children were given the visual search task instructions and asked to place their hands on the handprint outlines in front of the monitor. The task began with three blocks of practice trials, equivalent to those of LoBue and Deloache (2008). The first block involved six trials in which a single picture of a quoll, quokka, cuscus, or one set of leaves from each of the three distracter sets appeared on the screen alone, and the child was required to touch the picture as quickly as possible. The second block involved three trials showing one picture of a quokka, quoll, or cuscus positioned next to one of the corresponding piles of leaves, and children were asked to touch the quokka, quoll, or cuscus as quickly as possible. All pictures were randomly selected from the 24 photographs. The final block of four practice trials involved one X (target stimulus) among eight Ys (distracter stimuli), and the child was instructed to touch the X as quickly as possible. After children had completed the practice trials, they began the actual trials.

There were six types of trial in six blocks; these were one cuscus among eight piles of leaves; one pile of leaves among eight cuscuses; one quokka among eight piles of leaves; one pile of leaves among eight quokkas; one quoll among eight piles of leaves; and one pile of leaves among eight quolls. All children completed all trials, but the order of blocks was randomized. In each block, each of the 24 pictures was used as the target stimulus once, presented in one of the nine positions (randomly) in the matrix two or three times. The 24 distracter pictures appeared randomly and were presented multiple times, with each distracter being shown approximately the same number of times across trials. If children successfully touched the target stimulus, a large smiley face appeared in the center of the screen, with a picture of Safari Sam looking happy and the text “Correct! Well done!” If they touched one of the distracter stimuli, a sad-looking Safari Sam appeared on the screen with the instructions “Incorrect. But don’t worry – I found it for you! Be sure to look carefully but remember to be as quick as you can!’

After each trial, children replaced their hands back on the handprints, and once they were looking at the smiley face, the experimenter touched the screen in order for the next visual search matrix to appear. This ensured their hands were in the same position at the beginning of each trial, and also that their attention was on the screen prior to the matrix appearing. Response times were recorded automatically from the onset of the matrix to the time children touched a stimulus.

##### Behavioral avoidance task and heart rate response

In the touch box task (see also Askew & Field, 2007; Field & Lawson, 2003; Kelly et al., 2010), children were shown three pet-carrier boxes (size = 26 cm × 46 cm × 34 cm) and told that the boxes each contained one of the three animals. Each box had breathing holes around the top of the box, and one large round hole (diameter = 14 cm) covered in hessian for the child to place their hand in the box without being able to see into it, as well as a picture of a quokka, quoll, or cuscus on it so the children could easily differentiate which animal was supposedly in each box. The box was filled with straw and pieces of straw were poking through the hessian to add to the realism. All boxes were actually empty, and the children were informed of this and shown the empty box after completing the experiment. The boxes were positioned so that the quokka was on the left, the cuscus in the middle, and the quoll on the right. A line was marked on the floor with masking tape 1 m from the boxes; this was the starting point for each approach trial. Children were asked to stand on the line and then approach the first box on the experimenter’s instruction. The time it took to approach each box was recorded to indicate children’s approach–avoidance behavior. The stopwatch began as soon as a child had been asked to approach the first animal. After 15 s, regardless of whether children approached the box or not, they were instructed that the trial was over and asked to stand on the line opposite the next box. The procedure was the same for the final two boxes. Children were reminded that they did not have to touch the animal if they did not want to, and if a child made no attempt to approach the box within 15 s, they were judged to not wish to participate in that trial.

During the touch box task, children’s heart rate was measured via a Contec Finger Probe Pulse Oximeter in order to measure any physiological change associated with approaching the boxes they believed to contain the three animals. Children were asked to place the monitor on the index finger of their left hand (if they were happy to do so), and the experimenter checked that a stable reading was displayed before the touch box task commenced. Heart rate was measured at four time intervals: as the touch box task commenced (at 0 s), when the child approached the box, when the child put their hand in the box, and then when the child withdrew their hand. This small, portable device was chosen because it was nonintrusive and allowed children free movement to approach the boxes.

##### Nature reserve task

The nature reserve task (Field & Storksen-Coulson, 2007) was created on a 680 mm × 500 mm rectangular board, and embellished with green felt and pipe-cleaner trees and fences to add to the realism. The trees and fences were on the edges and corners of the board, so the children were unable to “hide” behind them. A picture of a quokka, cuscus, or quoll was positioned at one end of the board in three counterbalanced conditions. Children placed a Duplo figure representing themselves on the board where they would most rather be in relation to the animal. The distance between the animal and figure was measured in each case and taken as an indication of children’s approach–avoidance preferences for the animals.

### Procedure

Each child participated individually in a familiar school room, with no distractions, and took approximately 25 min. Children first completed the STAIC and the MASC-10 by hand. The automated procedure began with children completing the FBQ using the touch-screen monitor, followed by the vicarious learning procedure. They then completed the FBQ for a second time to establish whether fear beliefs had changed for the animals as a result of vicarious learning. Next, children completed the visual search task. They were then asked to consecutively approach each of the touch boxes (if they were happy to do so), and for each animal, their heart rate was measured at 0 s, when children approached the box, when they put their hand in the box, and when they withdrew their hand. In the final part of the experiment, children were given the nature reserve task. Children completed the FBQ, the visual search task, and the nature reserve task again 1 week later (Time 2) and 1 month later (Time 3). Follow-up measures were not taken for the touch box task because children were aware that there were no animals in the boxes after completing the task at Time 1. At the end of the final follow-up session, children were fully debriefed about the nature of the study and had an opportunity to ask questions. The aim of the research was explained verbally to children in age-appropriate language, including what the aims of the study were, why they were shown the pictures, why they were asked the questions, and why they were asked to do the tasks. In addition, in order to redress any false impressions that children may have had about the animals, they were given an information sheet containing true information about the animals and age-appropriate worksheets about the animals to complete.

## Results

A rejection criterion of alpha = .05 was used for all analyses. Effect sizes are reported as *r*, when interpretable, and otherwise as partial eta-squared (η_p_^2^). Cohen’s (1988, 1992) suggestions about what constitutes a large or small effect are as follows: *r* = .10 is a small effect, *r* = .30 is a medium effect, and *r* = .50 is a large effect. For partial eta-squared, η_p_^2^ = .02 is a small effect, η_p_^2^ = .13 is a medium effect, and η_p_^2^ = .26 is a large effect.

### Fear Beliefs

**Figure 1 fig1:**
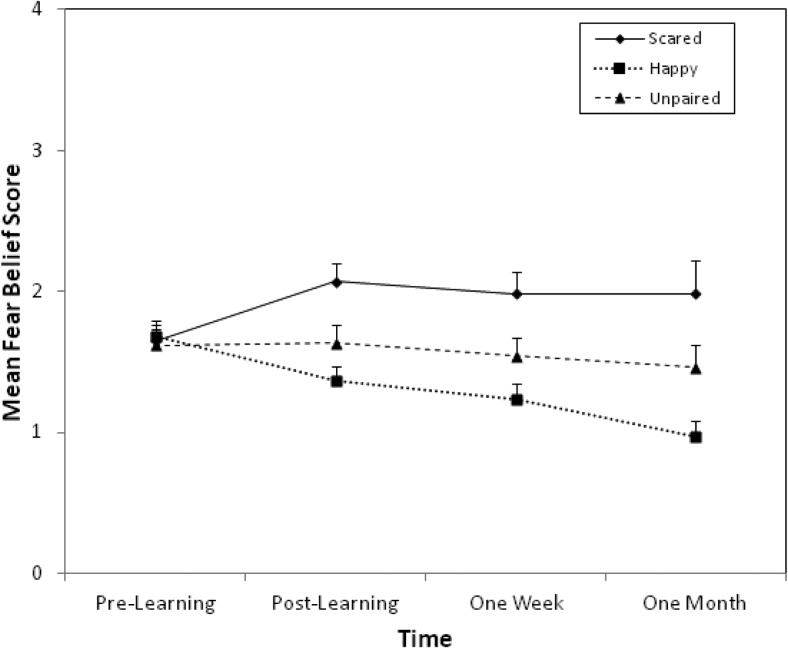
Mean (and *SE*) fear belief scores for scared-paired, happy-paired, and unpaired animals, before and after vicarious learning, 1 week later, and at 1-month follow-up.

Figure 1 displays mean fear belief scores before and after vicarious learning and at 1-week and 1-month follow-ups, for the scared-paired, happy-paired, and unpaired animals. The graph shows an increase in fear beliefs for the scared-paired animal compared to the unpaired animal, which remained at 1-week and 1-month follow-up, and a decrease in fear beliefs for the happy-paired animal compared to the unpaired animal.[Fig-anchor fig1]

A two-way 4 (time: prelearning, postlearning, 1 week, 1 month) × 3 (pairing type: scared-paired, happy-paired, unpaired) repeated measures ANOVA conducted on average fear belief scores for the three animals revealed no significant main effect of time, *F*(1.87, 67.35) = 1.49, *p* = .23, but a significant main effect of pairing type, *F*(2, 72) = 17.48, *p* < .001, η_p_^2^ = .33. The critical Time × Pairing Type interaction was significant, *F*(3.34, 120.11) = 6.85, *p* < .001. Planned comparisons compared differences between mean fear beliefs for scared-paired and unpaired animals at each time point to prelearning (baseline) differences in fear beliefs for these animals. Fear beliefs for scared-paired animals had increased significantly postlearning compared to the unpaired animal, *F*(1, 36) = 6.21, *p* = .02, *r* = .38, and remained elevated 1 week, *F*(1, 36) = 12.20, *p* = .001, *r* = .50, and 1 month, *F*(1, 36) = 4.28, *p* = .046, *r* = .33, later. Similar decreases in fear beliefs were found for happy-paired animals compared to the unpaired animal postlearning, *F*(1, 36) = 4.07, *p* = .051, *r* = .32, and remained lower at 1 week, *F*(1, 36) = 10.73, *p* = .002, *r* = .48, and 1 month, *F*(1, 36) = 11.17, *p* = .002, *r* = .49.

### Avoidance Preferences

**Figure 2 fig2:**
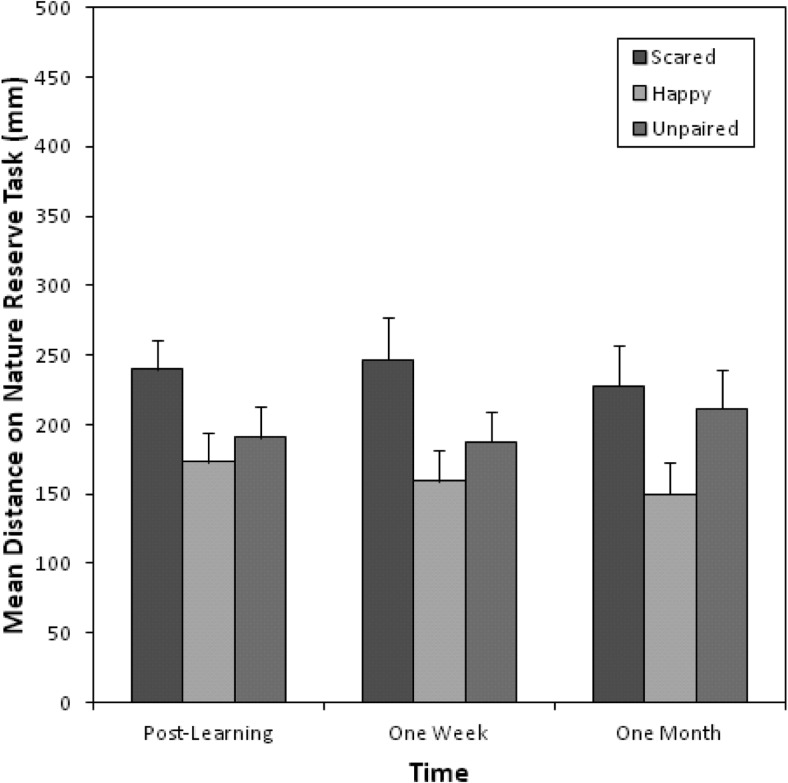
Mean distances (mm) from the scared-paired, happy-paired, and unpaired animal on the nature reserve task after vicarious learning, 1 week later, and at 1-month follow-up.

Figure 2 shows the mean distance children placed their figure from the scared-paired, happy-paired, and unpaired animals in the nature reserve task. Children placed themselves further away from scared-paired animals compared to happy-paired and unpaired animals at all three time points.[Fig-anchor fig2]

A two-way 3 (pairing-type: scared, happy, unpaired) × 3 (time: post learning, 1 week, 1 month) repeated measures ANOVA was used to analyze the distance measurements from the animals, indicating no significant main effect of time, *F*(2, 72) = 0.12, *p* = .89, η_p_^2^ = .003, but a significant main effect of pairing-type, *F*(2, 72) = 6.81, *p* = .002, η_p_^2^ = .16. Planned comparisons indicated significant differences in avoidance preferences between scared-paired animals compared to the happy-paired animals, *F*(1, 36) = 13.13, *p* = .001, *r* = .52. Differences between scared-paired and unpaired animals, *F*(1, 36) = 3.04, *p* = .09, *r* = .28, and happy-paired and unpaired animals, *F*(1, 36) = 3.99, *p* = .053, *r* = .32, approached, but did not quite reach, significance, though effect sizes were still small to medium (Cohen, 1988, 1992). There was no significant Pairing Type × Time interaction, *F*(4, 144) = 0.88, *p* = .48, η_p_^2^ = .02, indicating there was no significant difference in vicariously acquired avoidance preferences between different time points.

### Behavioral Avoidance

Children were given a maximum of 15 s to approach each touch box, so any child who chose not to take part in the touch box task had 15 s attributed as their approach time. Nine children chose not to approach the scared-paired animal, eight children chose not to approach the happy-paired animal, and seven children chose not to approach the unpaired animal. A repeated measures ANOVA performed on approach times indicated a significant main effect of pairing type (scared, happy, or unpaired), *F*(1.64, 70.33) = 3.62, *p* = .041. Planned comparisons comparing approach times for the animals revealed significantly longer approach times for scared-paired animals (*M* = 5.77, *SD* = 5.77) compared to unpaired animals (*M* = 4.55, *SD* = 5.55), *F*(1, 43) = 5.74, *p* = .02, *r* = .34, but no significant difference for happy-paired animals (*M* = 5.11, *SD* = 5.68) compared to unpaired animals, *F*(1, 43) = 2.18, *p* = .15, *r* = .22.

### Heart Rate

**Figure 3 fig3:**
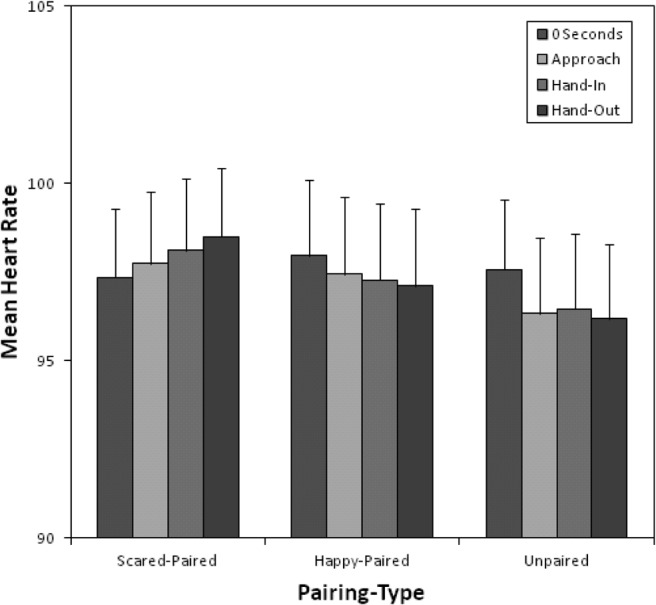
Mean heart rates (and *SE*) at 0 s, on approach, while hand is in the box, and when the hand is out the box, for scared-paired, happy-paired, and unpaired animals during the touch box task.

Figure 3 shows mean heart rates at baseline, as children approached the box, as they put their hand into the box, and as they withdrew their hand from the box. The means suggest an increase in heart rate when approaching the scared-paired animal, but very little change when approaching the happy-paired and unpaired animals.[Fig-anchor fig3]

A two-way 3 (pairing type: scared-paired, happy-paired, unpaired) × 4 (time: baseline, approach, hand-in, hand-out) repeated measures ANOVA revealed no significant main effect of pairing type, *F*(2, 68) = 1.19, *p* = .31, η_p_^2^ = .03, but a significant main effect of time, *F*(1.40, 47.73) = 6.65, *p* = .007, η_p_^2^ = .16, and, more important, a significant Time × Pairing interaction, *F*(3.50, 118.89) = 4.03, *p* = .006, η_p_^2^ = .11. Planned comparisons indicated that there was no significant change in children’s heart rate from baseline to approaching the box for scared-paired compared to unpaired animals, *F*(1, 34) = 3.11, *p* = .087, *r* = .29. However, children’s heart rate was significantly elevated compared to baseline as they put their hands in, *F*(1, 34) = 4.90, *p* = .034, *r* = .35, and out, *F*(1, 34) = 8.30, *p* = .007, *r* = .44, of the scared-paired animal boxes. In contrast, there were no significant changes in children’s heart rate at any point in the approach cycle for happy-paired compared to unpaired animals, from baseline to approaching the box, *F*(1, 34) < 0.01, *p* > .99, putting their hand in the box, *F*(1, 34) = 0.37, *p* = .55, *r* = .10, and withdrawing their hand from the box, *F*(1, 34) = 0.11, *p* = .74, *r* = .06.

### Attentional Bias

RTs less than 200 ms and all incorrect responses were excluded. Log RTs were used to adjust for large outliers (see Ratcliff, 1993). The number of incorrect responses was 5.87% postlearning, 7.42% at 1 week, and 5.92% at 1 month, and did not vary by target. Multilevel modeling was used to explore the prediction that children’s attentional bias for scared-paired animals increases proportionally with increases in fear beliefs. Time (0 days, 7 days, 30 days), pairing type (scared-paired, unpaired, happy-paired), and changes in fear beliefs compared to baseline were treated as being nested within the child, and the outcome variable was log RTs for detecting the animal.

**Figure 4 fig4:**
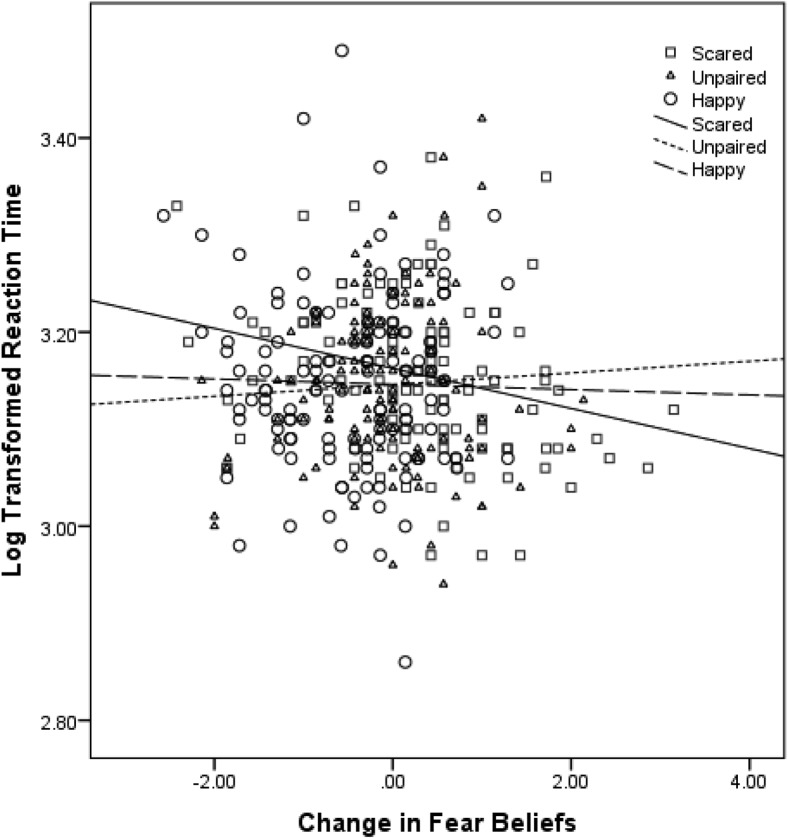
Log-transformed mean RTs and change in fear beliefs for scared-paired and unpaired animals after vicarious learning, 1 week later, and 1 month later.

The analysis indicated a significant main effect of time, *b* = −0.0009, *SE* = 0.0005, *t*(311) = −1.98, *p* = .048, and changes in fear beliefs, *b* = 0.03, *SE* = 0.01, *t*(311) = 2.44, *p* = .02. The main effect of scared-pairing type was approaching significance, *b* = 0.02, *SE* = 0.012, *t*(311) = 1.82, *p* = .070, but there was no significant main effect of happy-pairing type, *b* = 0.0004, *SE* = 0.012, *t*(311) = 0.04, *p* = .97. The Time × Change in Fear Beliefs interaction approached significance, *b* = −0.001, *SE* = 0.0006, *t*(311) = −1.87, *p* = .06. For the scared-paired animal, the important interaction between pairing type (scared vs. unpaired) and changes in fear beliefs was significant, *b* = −0.04, *SE* = .02, *t*(311) = −2.53, *p* = .01, showing that the length of time children took to detect animals was predicted by whether the animal had been seen with scared faces and moderated by the changes in their fear beliefs for that animal. Figure 4 indicates that when fear beliefs increased, differences in RTs for scared-paired verses unpaired animals were positive, but when fear beliefs decreased, differences were negative. That is, increases in fear beliefs caused by fear-related vicarious learning were associated with an attentional bias toward the animals; so children whose fear beliefs had increased the most were fastest at locating scared-paired animals compared to unpaired animals. Finally, the Time × Pairing Type interaction, *b* = −0.0007, *SE* = 0.0007, *t*(311) = −1.02, *p* = .31, and Time × Pairing Type × Changes in Fear Beliefs interaction, *b* = 0.001, *SE* = 0.0007, *t*(311) = 1.29, *p* = .20, were not significant, indicating that the effect did not change over time.[Fig-anchor fig4]

For the happy-paired animal, there was no significant Time × Pairing Type (happy paired vs. unpaired) interaction, *b* = 0.00005, *SE* = 0.0007, *t*(311) = 0.07, *p* = .94 or Time × Happy Pairing Type × Changes in Fear Beliefs interaction, *b* = 0.001, *SE* = 0.0008, *t*(311) = 1.35, *p* = .18. However, the Pairing Type × Changes in Fear Beliefs interaction was approaching significance, *b* = −0.03, *SE* = 0.02, *t*(311) = −1.93, *p* = .055. Figure 4 shows that detection times for happy-paired animals changed very little with increases and decreases in fear beliefs for these animals, but also suggests that the greater decreases in fear beliefs for happy-paired animals were, the slower detection times for these animals were compared to unpaired (control) animals.

A further multilevel modeling analysis was conducted on log RTs for detecting leaf stimuli among an array of scared-paired, happy-paired, or unpaired distracters to determine the effect of vicarious learning on attentional bias postlearning, at 1 week, and at 1 month. The analysis indicated a significant main effect of time, *b* = −0.001, *SE* = 0.0004, *t*(311) = −3.08, *p* = .002, and scared-pairing type, *b* = 0.02, *SE* = 0.01, *t*(311) = 2.15, *p* = .03, but the main effect of changes in fear beliefs, *b* = −0.006, *SE* = 0.01, *t*(311) = −0.53, *p* = .60, and happy-pairing type, *b* = −0.01, *SE* = 0.01, *t*(311) = −1.02, *p* = .31, were nonsignificant. The Time × Change in Fear Beliefs interaction was also not significant, *b* = −0.0001, *SE* = 0.0005, *t*(311) = −0.36, *p* = .72. For the scared paired animal, there was no significant Time × Pairing type (scared paired vs. unpaired) interaction, *b* = −0.0005, *SE* = 0.0006, *t*(311) = −0.87, *p* = .39, Pairing Type × Changes in Fear Beliefs interaction, *b* = −0.02, *SE* = 0.01, *t*(311) = −1.35, *p* = .18, or Time × Pairing Type × Changes in Fear Beliefs interaction, *b* = 0.0009, *SE* = 0.0006, *t*(311) = 1.46, *p* = .14. For the happy paired animal, there was no significant Time × Pairing Type (happy paired vs. unpaired) interaction, *b* = 0.0004, *SE* = 0.0006, *t*(311) = 0.59, *p* = .55, Pairing Type × Changes in Fear Beliefs interaction, *b* = −0.02, *SE* = 0.01, *t*(311) = −1.09, *p* = .28, or Time × Pairing Type × Changes in Fear Beliefs interaction, *b* = 0.001, *SE* = 0.0007, *t*(311) = 1.48, *p* = .14.

### Trait Anxiety

**Figure 5 fig5:**
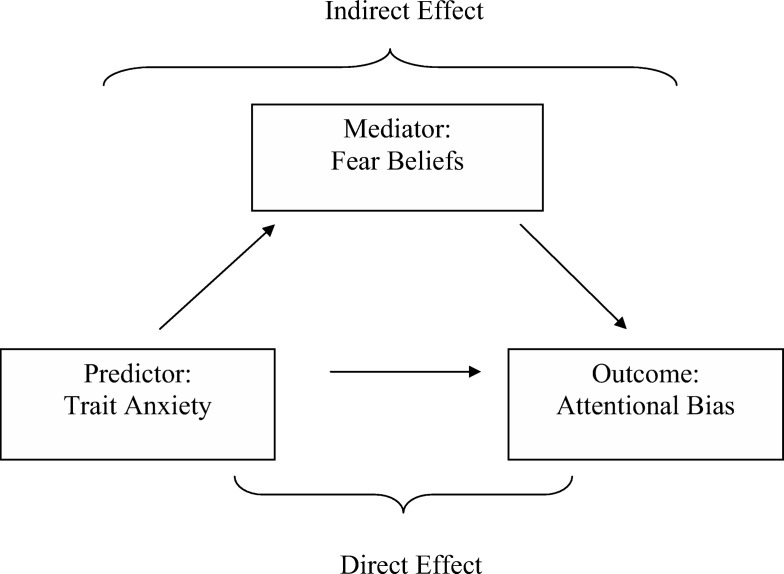
Mediation analysis to determine whether trait anxiety predicts attentional bias mediated by changes in fear beliefs.

A Hayes-style (2013) mediation analysis was used to explore the second hypothesis that higher trait anxiety leads to greater attentional bias, and this is mediated by changes in fear beliefs (see Figure 5 for a diagrammatic representation). An attentional bias score was first calculated by subtracting log transformed RTs for detecting the scared-paired animal from the log transformed RTs for detecting the unpaired animal. The resulting score is an indication of how much faster children were at detecting scared paired compared to unpaired animals.[Fig-anchor fig5]

Trait anxiety as measured using the STAIC did not significantly predict either postlearning changes in fear beliefs for scared-paired animals, *b* = 0.01, *SE* = 0.02, *t*(42) = 0.70, *p* = .49, or postlearning attentional bias for scared-paired animals, *b* = −0.0008, *SE* = 0.002, *t*(42) = 0.35, *p* = .73. Postlearning changes in fear beliefs also did not significantly predict attentional bias for scared-paired animals, *b* = −0.0001, *SE* = 0.02, *t*(42) = 0.003, *p* = .99. At 1 week, STAIC scores significantly predicted attentional bias for scared-paired animals, *b* = −0.003, *SE* = 0.001, *t*(39) = −2.23, *p* = .03, but not changes in fear beliefs for these animals, *b* = 0.007, *SE* = 0.02, *t*(39) = 0.38, *p* = .71. Changes in fear beliefs for the scared-paired animal did not significantly predict attentional bias, *b* = 0.01, *SE* = 0.01, *t*(39) = 0.96, *p* = .34. At 1 month, STAIC scores did not significantly predict change in fear beliefs for scared-paired animals, *b* = 0.03, *SE* = 0.04, *t*(35) = 0.66, *p* = .51, nor did trait anxiety predict attentional bias for these animals, *b* = −0.002, *SE* = 0.002, *t*(35) = −1.21, *p* = .24; changes in fear beliefs also did not predict attentional bias for scared-paired animals, *b* = 0.007, *SE* = 0.007, *t*(35) = 1.04, *p* = .30. Thus, there was no indication of any relationship between STAIC scores and attentional bias for scared-paired animals, except at 1 week following vicarious learning, but this relationship was not mediated by changes in fear beliefs.

For happy-paired animals, STAIC scores did not significantly predict either postlearning changes in fear beliefs, *b* = 0.01, *SE* = 0.02, *t*(42) = .70, *p* = .49, or postlearning attentional bias for happy-paired animals, *b* = 0.0006, *SE* = 0.002, *t*(42) = 0.28, *p* = .78. Postlearning changes in fear beliefs also did not significantly predict attentional bias for happy-paired animals, *b* = −0.02, *SE* = 0.02, *t*(42) = 1.12, *p* = .27. At 1 week, STAIC scores did not significantly predict changes in fear beliefs for happy-paired animals, *b* = −0.01, *SE* = 0.02, *t*(39) = −0.62, *p* = .54, or attentional bias for happy-paired animals, *b* = 0.0007, *SE* = 0.002, *t*(39) = 0.44, *p* = 66. Changes in fear beliefs for the happy-paired animal did not significantly predict attentional bias, *b* = −0.005, *SE* = 0.02, *t*(39) = −0.28, *p* = .78. At 1 month, STAIC scores did not significantly predict change in fear beliefs for happy-paired animals, *b* = −0.007, *SE* = 0.02, *t*(35) = 0.36, *p* = .72, or attentional bias for these animals, *b* = 0.0005, *SE* = 0.002, *t*(35) = 0.32, *p* = .75. Changes in fear beliefs also did not predict attentional bias for happy-paired animals, *b* = 0.009, *SE* = 0.01, *t*(35) = 0.74, *p* = .46. Thus, there was no indication of any relationship between STAIC scores and attentional bias for happy-paired animals.

The same analyses were conducted using MASC scores as a measure of children’s general anxiety levels. MASC scores significantly predicted changes in fear beliefs for scared-paired animals immediately following vicarious learning, *b* = 0.03, *SE* = 0.009, *t*(42) = 3.28, *p* = .002, but not attentional bias for them, *b* = −0.002, *SE* = 0.002, *t*(42) = −1.04, *p* = .31. Changes in fear beliefs for scared-paired animals also did not predict attentional bias for these animals following vicarious learning, *b* = 0.01, *SE* = 0.02, *t*(42) = 0.43, *p* = .67. At 1 week, MASC scores significantly predicted changes in fear beliefs for scared-paired animals, *b* = 0.02, *SE* = 0.01, *t*(39) = 2.04, *p* = .048, but not attentional bias, *b* = −0.0004, *SE* = 0.001, *t*(39) = 0.42, *p* = .67. Changes in fear beliefs did not predict a change in attentional bias for the scared-paired animal, *b* = 0.01, *SE* = 0.01, *t*(39) = 0.87, *p* = .39. At 1 month, MASC scores significantly predicted changes in fear beliefs for scared paired animals, *b* = 0.06, *SE* = 0.02, *t*(35) = 2.48, *p* = .02, and the relationship with attentional bias was approaching significance, *b* = −0.002, *SE* = 0.001, *t*(35) = 1.86, *p* = .07, but changes in fear beliefs did not significantly predict attentional bias for scared-paired animals, *b* = 0.01, *SE* = 0.007, *t*(35) = 1.59, *p* = .12. Thus, there was evidence that children’s anxiety as measured using the MASC-10 was related to attentional bias at 1 month, but not at any other time. There was no evidence that this relationship was mediated by changes in fear beliefs for scared-paired animals.

MASC scores did significantly predict changes in fear beliefs for happy-paired animals immediately following vicarious learning, *b* = 0.03, *SE* = 0.009, *t*(42) = 3.28, *p* = .002, showing that high trait anxiety was associated with lower decreases in fear beliefs for the happy-paired animal. However, MASC scores did not significantly predict attentional bias for the happy-paired animals, *b* = 0.0003, *SE* = 0.002, *t*(42) = 0.22, *p* = .82. Changes in fear beliefs for happy-paired animals also did not predict attentional bias for these animals following vicarious learning, *b* = −0.02, *SE* = 0.02, *t*(42) = 1.08, *p* = .29. At 1 week, MASC scores did not significantly predict changes in fear beliefs for happy-paired animals, *b* = −0.01, *SE* = 0.01, *t*(39) = 1.11, *p* = .27, or attentional bias, *b* = −0.0004, *SE* = 0.001, *t*(39) = 0.42, *p* = .68. Changes in fear beliefs did not predict a change in attentional bias for the happy-paired animal, *b* = −0.006, *SE* = 0.02, *t*(39) = 0.39, *p* = .70. At 1 month, MASC scores did not significantly predict changes in fear beliefs for happy paired animals, *b* = −0.01, *SE* = 0.01, *t*(35) = −1.01, *p* = .32, or attentional bias, *b* = −0.0002, *SE* = 0.001, *t*(35) = 0.25, *p* = .81, and changes in fear beliefs did not significantly predict attentional bias for happy-paired animals, *b* = 0.009, *SE* = 0.01, *t*(35) = 0.67, *p* = .51. Thus, there was no indication of any relationship between MASC scores and attentional bias for happy-paired animals.

### Correlation Analysis

Correlation analyses were carried out in order to explore whether there was any concordance between the various measures of fear—self-report, behavioral, and physiological—because previous work has failed to demonstrate a strong relationship between different types of fear response in adults (e.g., Hodgson & Rachman, 1974; Lang et al., 1970; Rachman & Hodgson, 1974; Zinbarg, 1998). Children’s fear beliefs were significantly correlated with avoidance preferences following learning, *r*(42) = .40, *p* = .008, at 1 week, *r*(39) = .53, *p* < .001, and again at 1 month, *r*(36) = .43, *p* = .007. However, there was no significant relationship between children’s postlearning fear beliefs and behavioral avoidance, *r*(33) = .25, *p* = .15, or heart rate when approaching touch boxes, *r*(33) = .26, *p* = .14, though correlations with heart rate when withdrawing their hands from touch boxes approached significance, *r*(33) = .29, *p* = .09. The correlation between children’s fear beliefs and their heart rate when putting their hands in the boxes also approached significance, *r*(33) = .30, *p* = .08. Fear beliefs and attentional bias were not significantly correlated following learning, *r*(42) = −.18, *p* = .24, or at 1 month, *r*(35) = −.23, *p* = .17; however, they were significantly correlated at 1 week, *r*(39) = −.41, *p* = .007.

Following learning, children’s avoidance preferences were not significantly correlated with behavioral avoidance, *r*(35) = .07, *p* = .70, heart rate when approaching the animal, *r*(33) = .001, *p* = .99, heart rate when putting their hands in the box, *r*(33) = .04, *p* = .81, or heart rate when withdrawing their hands, *r*(33) = .04, *p* = .82. Avoidance preferences were not significantly correlated with attentional bias following learning, *r*(42) = −.01, *p* = .94, at 1 week, *r*(39) = −.20, *p* = .22, or at 1 month, *r*(35) = −.06, *p* = .73.

Children’s behavioral avoidance on the touch box task was (borderline) significantly related to their heart rate when withdrawing their hands from boxes, *r*(33) = .33, *p* = .053. Correlations between behavioral avoidance and heart rate when approaching boxes, *r*(33) = .29, *p* = .088, and when putting their hand in the boxes, *r*(33) = .32, *p* = .06, were approaching significance. No correlation was found between children’s behavioral avoidance and attentional bias following learning, *r*(33) = .16, *p* = .36. Finally, attentional bias following learning was not significantly correlated with heart rate when approaching boxes, *r*(33) = .15, *p* = .38, heart rate when putting their hands in the boxes, *r*(33) = .15, *p* = .38, or heart rate when withdrawing their hand from boxes, *r*(33) = .15, *p* = .40.

In summary, self-reported cognitions correlated significantly with avoidance preferences at all three time points and for some heart rate measures. Behavioral avoidance also showed moderate correlation with heart rate following learning. All remaining correlations were nonsignificant. Thus, results indicate mixed evidence for correlations between different fear response systems in children.

## Discussion

The current study investigated whether vicarious fear learning can create physiological responses and attentional bias in children. Results showed an increase in self-reported fear-beliefs, and avoidance behavior following vicarious learning, providing further support for vicarious learning as a pathway to fear acquisition during childhood. Moreover, changes in fear beliefs and avoidance preferences were still present at follow-up 1 week and 1 month later, supporting previous longitudinal evidence demonstrating the robustness of vicariously learnt responses (Askew & Field, 2007). Thus, the current findings support studies (Askew & Field, 2007; Askew et al., 2008) showing that vicarious learning causes changes in two of Lang’s (1968) anxiety response systems: language behavior (subjective-report) and overt behavior (avoidance). Additionally, the experiment explored the effect of vicarious learning on Lang’s third and final response system: physiological changes. Results showed an increase in heart rate following fear-related vicarious learning, before and after children put their hand in a box they believed contained their scared-paired animal.

Finally, the experiment also investigated whether fear-related vicarious learning creates an attentional bias for novel animals. Findings indicated that the effect of vicarious learning on detection times for scared-paired animals was moderated by increases in fear beliefs for these animals during learning: larger increases in fear beliefs led to greater attentional bias. Thus, this represents the first experimental evidence showing that vicarious fear-learning can create an attentional bias toward animals. The expected relationship between trait anxiety (STAIC) and attentional bias for scared-paired animals was found only at 1-week follow-up, and this relationship was not mediated by changes in fear beliefs, as predicted. On the other hand, there was evidence that children’s anxiety as measured using the MASC-10 was related to attentional bias at 1 month, but not at any other time. Again, this relationship was not mediated by changes in fear beliefs for scared-paired animals.

Previous research has demonstrated changes in self-reported fear beliefs, avoidance (Field & Lawson, 2003), and heart rate (Field & Schorah, 2007), as a result of Rachman’s (1977) other indirect pathway: threat-related verbal information. Studies have also shown that vicarious learning leads to increases in self-reported fear beliefs (e.g., Askew & Field, 2007) and that self-reported fear is associated with increased heart rate in feared situations (e.g., E. W. Cook et al., 1988; Lang, 1971; Lang et al., 1970; Sartory et al., 1977; Weems et al., 2005). But this is the first experiment to directly establish that fear-related vicarious learning increases children’s heart rate responses for stimuli. Thus the findings represent the first evidence in children that vicarious learning leads to changes in all three of Lang’s (1968) anxiety systems. Heart rate responses are likely to be under less conscious control than cognitions and behavior, and are therefore less prone to demand characteristics. Thus evidence of changes in all three response systems provides compelling experimental support for the vicarious learning pathway to fear acquisition during childhood.

Changes in fear cognitions and avoidance have been demonstrated in previous research (e.g., Askew & Field, 2007; Gerull & Rapee, 2002), but there is not always correlation between physiological changes and these measures (Hodgson & Rachman, 1974; Lang et al., 1970; Rachman & Hodgson, 1974; Zinbarg, 1998). The current study showed a relationship between some of the fear response systems in children, but not others. Specifically, children’s avoidance preferences correlated with their fear beliefs but not with their heart rate responses; behavioral avoidance in the touch box task was borderline significantly related to children’s heart rate when withdrawing their hands from boxes; and correlations between behavioral avoidance and heart rate when approaching boxes and when putting their hand in the boxes approached significance. Finally, although not statistically significant here, correlations between heart rate and fear beliefs were similar in magnitude to significant associations between heart rate and measures of anxiety and anxiety control beliefs in a recent study by Scott and Weems (2014). The researchers used a more specific measure of heart rate—vagal tone—than the one used here, and it is possible that more subtle measures of heart rate such as this show a clearer relationship to other measures in the anxiety response system.

Studies of anxious adults have indicated information processing biases of attention, reasoning, and memory directed at threatening stimuli (Cisler & Koster, 2010; Hadwin, Garner, & Perez-Olivas, 2006; Harvey, Watkins, Mansell, & Shafran, 2004). Research with anxious children shows that attentional biases can also be detected in children (DeLoache & LoBue, 2009; LoBue, 2009; LoBue & DeLoache, 2008, 2010; see Hadwin & Field, 2010, for a review), indicating, in particular, that anxious children attend disproportionately to threatening stimuli (Garner, 2010; Heim-Dreger, Kohlmann, Eschenbeck, & Burkhardt, 2006). The current findings support previous research using visual search tasks that has frequently demonstrated that clinically anxious individuals display an attentional bias toward threat (e.g., Hadwin et al., 2003; Miltner et al., 2004; Rinck et al., 2003; Rinck et al., 2005).

Field and Lester (2010b) have suggested a moderation model in which information biases are maintained in high-trait-anxious children but decline in low-anxious children with increasing age. In accordance with the predictions of Field and Lester’s model, it was found that high-anxious children showed greater attentional bias at 1-week (measured by the STAIC) and 1-month follow-ups (measured by the MASC). However, a relationship between STAIC or MASC-10 scores and RTs was not found at any other time points; therefore, support for this model was equivocal at best. Past evidence does support this relationship (see, e.g., Field et al., 2011; Hadwin & Field, 2010), and an alternative explanation for the findings may be that they reflect methodological differences. The most obvious of these is the use of a touch-screen monitor to measure attentional biases. Responding using a touch screen involves recognizing that a target is present, identifying its location, and physically touching that part of the screen. This may require more conscious effort than merely pressing a key to identify a target is present, which is likely to involve automatic processing, and this method may not be sensitive enough to detect the relationship between attention and anxiety levels.

The vicarious learning in the study was very mild compared with real-life observational fear learning leading to phobias, because, for obvious ethical reasons, the images (emotional faces and animals) used in the procedure did not constitute anything children in this age group could not come across in their everyday lives anyway. Thus, like Askew and Field (2007), the avoidance behavior here is mild compared with real-life phobic avoidance. But these increases following learning are important for two reasons. First, even mild behavioral and attentional avoidance of animals could theoretically maintain phobias by preventing fear beliefs from being disconfirmed. In addition, vicarious learning events occurring outside of the laboratory are likely to be more intense than those experienced in the current procedure, involving auditory information and moving animals and models. Therefore, we might expect learned responses to be magnified compared with those in the laboratory. Indeed, one advantage of the current research is that the procedure represents a relatively benign way to study fear-related vicarious learning in children. Thus, it can be used as a nonharmful analogue of learning events in the real world to investigate potential preventions, early interventions, and methods of unlearning fear (e.g., Dunne & Askew, 2013).

One possible limitation of the procedure was that it was not possible to test whether avoidance and heart-rate effects were maintained for 1 week and 1 month. This was because children were aware that the touch boxes were empty after they had put their hands in all three boxes, so this task could not be repeated at 1-week and 1-month follow-ups. The study did, however, show that fear beliefs remained elevated for the entire 1 month period, and effect sizes for avoidance preferences were reasonable (.28 and .32), though not quite reaching conventional significance for this sample size. Nevertheless, one possibility is that behavioral avoidance stems directly from these fear-related beliefs and preferences, so that behavioral avoidance may have also remained fairly constant during this time. One way to investigate this in the future would be to conduct the touch box task for the first time either at 1-week or 1-month follow-up.

Another potential limitation is that although differences in ratings of brightness for animal targets and leaf distracters in the visual search task were nonsignificant, effect sizes varied from small to large (.19, .49, and .62), suggesting differences in stimulus salience that did not reach significance because of the small sample size (*N* = 6). However, response times for targets and distractors were made within each child, and animals were counterbalanced across children so that, effectively, three counterbalancing groups of children had seen each of the three animals with a different emotional face. Therefore, although the differences in saliency were unfortunate, this should not have affected comparisons of attentional bias.

To summarize, given the detrimental effects of anxiety for children and adults, understanding the processes of fear acquisition is fundamental in order to develop effective interventions. Understanding how and why vicarious learning occurs, and what its effects are on the wider fear emotion (e.g., physiological responses), can help us find more effective ways to prevent and reverse fears. The current study is important in broadening our understanding of the effects of vicarious learning by showing that fear-related vicarious learning results in (a) increases in children’s self-reported fear beliefs for novel animals, which remain elevated for at least 1 month; (b) behavioral avoidance of animals following learning; (c) increased heart-rate responses to the animals; and (d) attentional bias toward animals following vicarious learning.
